# Comparison of Biomedical Variables in PCOS Patients with Normal Iranian Women

**Published:** 2015-03

**Authors:** Maryam Bagheri, Farnaz Sohrabvand, Mahnaz Lankarani, Zahra Zandieh, Fedyeh Haghollahi, Mamak Shariat

**Affiliations:** 1 Reproductive Health Research Center, Tehran University of Medical Sciences, Tehran, Iran; 2 Endocrinology and Metabolism Research Center, Tehran University of Medical Sciences, Tehran, Iran; 3 Department of Anatomy, School of Medicine Sciences, Iran University of Medical Sciences, Tehran, Iran; 4 Maternal-Fetal and Neonatal Health Research Center, Tehran University of Medical Sciences, Tehran, Iran

**Keywords:** Female infertility, CRP, Polycystic ovary syndrome (PCOS)

## Abstract

**Objective:**To compared serum CRP levels and biochemical relation in PCOS patients with normal Iranian women.

**Materials and methods:**This case-control study was performed on 52 individuals with PCOS (Rotterdam 2003 criteria). The cases were compared to 104 healthy non-PCOS, 20 to 35-year-old female subjects with no history of diabetes or renal diseases. Blood samples were taken on the 2^nd^ to the 5^th^ day of menstrual cycle for the evaluation of CRP levels, triglyceride, insulin, androstronedion, testestrone and total cholesterol.

**Results:**The mean CRP was 1.38 (± 0.43) mg /dl in the PCOS group, and 1.08 (± 0.49) mg /dl (p= 0.240) in control group.High-Sensitivity C-Reactive Protein (HS-CRP) was positively correlated with the Body Mass Index (BMI) (r = 0.36, p= 0. 001). Before adjusting for age and BMI, CRP was correlated with LDL (r= 0.16, p= 0.03), total cholesterol (TC) (r= 0.17, p= 0. 03), Triglycerid (TG) (r= 0.23, p= 0.003), and the insulin (r= 0.20, p= 0. 01) notably in PCOS group. However, after adjustment was made for age and BMI, the correlation was attenuated in PCOS. The regression analyses depicted that CRP level was not under the influence of other medical parameters

**Conclusion:**The results showed that mean CRP level was not significantly different between PCOs and normal women**.** After adjustment for age and BMI, CRP was not associated with any biochemical marker evaluated in this study**.** It seems that studied biochemical serum levels were mostly associated with obesity. So reduction of BMI may normalize the serum levels of CRP and other biochemical parameters.

## Introduction

Polycystic ovary syndrome (PCOS) is found in 5-10% of women in the reproductive age (1). Its common clinical manifestations are insulin resistance, hyperandrogenism, anovulation and consequently, infertility. Affected women are also at higher risk of developing diabetes mellitus, artherosclerosis and cardiovascular disease (1-4) that is marked by abdominal obesity, insulin resistance, dyslipidemia, and atherosclerosis (5).

In addition women with PCOS have the sign of dyslipidemia, including increased plasma triglycerides, cholesterol, None Sterified Fatty Aacids (NEFA), low-density lipoprotein cholesterol (6-9), and markers of abnormal vascular function (10-12).

C-reactive protein (CRP), a low-grade chronic inflammatory factor, is a g-globulin that is closely linked to an increase in cardiovascular risk and it is an independent cardiovascular risk factor (13).

Numerous large-scale prospective studies have recognized that CRP is a strong independent predictor of future Cardiovascular Disease (CVD) and/or stroke (14,15). Previous studies have established that themeasurement of CRP compared with screening based on lipid levels may provide an improved method of identifying women at risk for CVD (16). These highly reliable clinicaldata are supported by many laboratory and experimental data demonstrating that atherothrombosis, in addition to being a disease of lipid accumulation, also representsa chronic inflammatory process (17).

C-reactive protein, especially its activated form in the blood vessel wall (18, 19), stimulates the expression of various adhesion molecules in the endothelium. Those molecules accelerate vascular inflammatory reactions and may accelerate the development of atherosclerosis (20, 21).

Therefore, considering the CRP possible ability to predict CVD, it seems that the healthy women with normal or high LDL Cholesterol (LDL-C) are prone to cardiovascular morbidity and mortality. Therefore, CRP may be an ideal marker for screening of apparently healthy young PCOS patients.

The question remains controversial of whether CRP levels are normal or increased in women with PCOS. An increased incidence of high levels of CRP in PCOS patients compared with controls (22, 23). Some studies have shown that CRP was elevated compared with age- and body mass index (BMI)–matched controls (24- 26). To confirm or rebute such an association, theCRP levels in the PCOS patients were measured and compared with the BMI-matched controls. Moreover, the lipid profiles in Iranian subjects with PCOS were investigated.

## Materials and methods

A case-control, cross-sectional study was designed to compare the levels of CRP in a group of PCOS patients and the control group. This study was performed from August 2010 through December 2011on 52 PCOS patients (case group) and 104 non PCOS patients (control group) and after obtaining the approval from the ethical committee of Tehran University of Medical Sciences. 

The inclusion criteria consisted of the patients with the age group of 20-35 years.

The patients were diagnosed with PCOS according to the revised 2003 criteria of the Rotterdam Criteria (27) that was defined as menstrual irregularity due to oligo menorrhea (fewer than nine menstrual periods per year) or amenorrhea (no menstrual periods for 3 or more months) and clinical evidence of hyper androgenism (hirsutism, acne, or male pattern balding). All women (those with PCOS and controls) were nonsmokers and were not taking any hormonal or insulin-modifying therapy or any other therapies that could affect metabolism, reproduction, or inflammation. (hormonal contraceptives, aspirin, statins, or any other medication for at least 2 months before blood examination). On the basis of interviews, none of the subjects had any known disease, including diabetes, cardiovascular disease, thyroid disease, or current infectious disease or ever had been pregnant (lifetime parity, zero). A glucose tolerance test was not performed on the subjects in either group. Fasting samples were assayed for the glucose, triglycerides, cholesterol, insulin, C-reactive protein (CRP), and the serum folate. Blood samples were drawn from an antecubital vein after an overnight fast, for testing of the blood glucose and insulin. The samples were processed by the centrifuge, and the plasma was aliquoted and stored at 20˚C until the analysis.

The plasma triglycerides and cholesterol were measured by using the Enzymatic (Pars Azmoon Kit, Iran). The insulin was measured by the electro immunoassay (DRG, Germany). C-reactive protein (CRP) was measured by the nephelometry (Orion Diagnostica Turbox, Finland). The normal concentration of CRP in the healthy human serum is usually lower than 10 mg/L.The body mass index was calculated as weight (kg)/height (m^2^). The insulin resistance index was calculated by using the homeostasis model assessment, computed with the following formula, as described elsewhere by Matthews et al.: [plasma glucose (mmol/L)]_ [serum insulin (mU/L)] (28).

The normal insulin sensitivity was defined on the basis of the fasting serum glucose and insulin levels. One of the indirect methods for the assessment of insulin resistance is Quicki (quantative insulin sensitivitycheck index) (29, 30). 

HOMA and Quicki indexes are calculated by using both the Fasting Insulin (FI) and the fasting blood glucose levels. Also FI ≥ 12 Mu/li have been proposed as the limiting level for IR, in non diabetic and diabetic population (30).

The serum insulin responses to an Oral Glucose Tolerance Test (OGTT) and the Homeostatic Model Of Insulin Resistance (HOMA-IR) (31, 32). HOMA-IR was calculated by the formula:

HOMA-IR = fasting blood sugar mg/dL× fasting insulin IU/mL/405 (33, 34).

A standard 75-g serum insulin response to OGTT and a test of the insulin response to the oral glucose loading were performed after 10–12 h of fasting, between 8:30 and 10:30 a.m. 

The baseline characteristics of the groups were presented as the mean ± standard deviation (SD). The laboratory parameters of the patients were compared by Student’s t test. The data were analyzed with SPSS (version 16, SPSS, Inc., Chicago, IL) for Windows; p ≤ 0.05 was considered statistically significant. The measured parameters in the two groups (PCOS vs. controls) were compared by the unpaired t test. The correlations of the parameters in the two groups were examined using the chi^2^ test. The linear correlations between the clinical parameters were assessed within each group by the Pearson correlation. In order to remove the confounding effect of the age and BMI, partial correlations were used.

## Results

The mean age of the PCOS group was 24.27 (3.75) yr, and in control group was 25.62 (4.3) yr. Baseline characteristics of the PCOS patients and the control group are summarized in Table 1. PCOS patients had significantly higher levels TG, androstenedion, and testestrone levels than the control group. PCOS had a higher BMI and were more insulin resistant than the healthycontrols.

There were no differences in terms of age, FBS, Cholestrole, LDL, and CRP between the groups. 

The mean CRP in the PCOS group was 1.38 (1.43) mg /dl, and in control group was 1.08 (1.49) mg /dl that is reported no difference between two groups (p=0.240).

HS-CRP) was positively correlated with the Body Mass Index (BMI) (r = 0.36, p= 0. 001). Before adjusting for age and BMI, CRP was correlated with LDL (r= 0.16, p= 0/03), total cholesterol (TC) (r= 0.17, p= 0. 03), Triglycerid (TG) (r= 0.23, p= 0.003), and the insulin (r= 0.20, p= 0. 01) notably in PCOS group (Table 2). However, after adjustment was made for age and BMI, the correlation was attenuated in PCOS (Table 3). The regression analyses depicted that CRP level was not under the influence of other medical parameters.

**Table 1:** Overall Clinical, endocrine and metabolic characteristics results in patients with PCO and Control group

	**PCO (Mean ± SD)** **n = 52**	**Control (Mean ± SD)** **n = 104**	**p value**
Age	24.27 (3.753)	25.62 (4.318)	0.057
BMI (kg/m2)	25.80 (6.672)	22.60 (2.873)	0.002
Homocysteine	12.21 (4.553)	13.68 (4.307)	0.057
Insulin	16.62 (7.453)	12.04 (4.239)	< 0.001
F.B.S (mmol/L)	87.50 (8.356)	86.00 (7.878)	0.274
Androstenedion	3.07 (1.520)	1.91 (1.042)	< 0.001
Testosterone	0.88 (.342)	0.71 (0.300)	0.001
Creatinin	0.90 (0.124)	0.87 (0.077)	0.076
Cholesterol (mmol/L)	1.6475E2 (37.726)	1.6070E2 (34.864)	0.507
LDL/C	92.63 (23.127)	88.87 (22.706)	0.334
HDL/C	49.42 (9.037)	53.10 (12.223)	0.057
Triglyceride (mmol/L)	1.1662E2 (73.020)	88.00 (50.299)	0.013
FAI	15.69 (29.421)	5.97 (6.462)	0.022
HOMA	33.72 (14.736)	44.56 (16.937)	< 0.001
QUIKI	0.32 (0.020)	0.34(.018)	< 0.001
G.I.R	6.22 (2.606)	7.99 (2.772)	< 0.001
CRP (mg/L)	1.38 (1.43)	1.08 (1.49)	0.240

**Table 2:** Simple Correlation between CRP and Age, BMI and Biomedical variables in PCO and Control group

	**Correlation coefficient (p-value)**		
	**PCO (n= 52)**	**Control (n= 104)**	**total (n= 156)**
**Age**	**0.137 (0.334)**	**0.043 (0.667)**	**0.055 (0.498)**
**BMI**	**0.542 (<0.001)**	**0.249 (0.011)**	**0.369 (<0.001)**
**INSULIN**	**0.289 (0.038)**	**0.107 (0.282)**	**0.203 (0.011)**
**F.B.S**	**-0.002 (0.987)**	**-0.054 (0.584)**	**-0.028 (0.728)**
**Androstenedion**	**-0.136 (0.337)**	**-0.129 (0.193)**	**-0.080 (0.324)**
**Testosterone**	**-0.139 (0.327)**	**-0.107 (0.282)**	**-0.089 (0.268)**
**Cholesterol**	**0.339 (0.014)**	**0.083 (0.402)**	**0.174 (0.030)**
**LDL/C**	**0.336 (0.015)**	**0.077 (0.435)**	**0.168 (0.036)**
**HDL/C**	**-0.190 (0.178)**	**-0.064 (0.518)**	**-0.109 (0.175)**
**Triglyceride**	**0.347 (0.012)**	**0.146 (0.139)**	**0.238 (0.003)**
**FAI**	**0.098 (0.491)**	**0.052 (0.599)**	**0.087 (0.282)**
**HOMA**	**-0.235 (0.093)**	**0.004 (0.964)**	**-0.091 (0.258)**
**QUIKI**	**-0.238 (0.090)**	**-0.024 (0.964)**	**-0.123 (0.125)**
**G.I.R**	**-0.249 (0.075)**	**-0.026 (0.793)**	**-0.118 (0.141)**
**Homocysteine **	**-0.010 (0.945)**	**-0.005 (0.960)**	**-0.021 (0.792)**

**Table 3:** Age and BMI adjusted correlation coefficient betweenCRP and Bio-medical variables

	**Correlation coefficient (p-value)**		
	**PCO (n= 52)**	**Control (n= 104)**	**total (n= 156)**
**Insulin**	**0.017 (0.905)**	**0.075 (0.457)**	**0.053 (0.516)**
**F.B.S**	**-0.082 (0.572)**	**-0.047 (0.638)**	**-0.056 (0.495)**
**Androstenedion**	**-0.178 (0.216)**	**-0.104 (0.302)**	**-0.124 (0.126)**
**Testosterone**	**-0.206 (0.150)**	**-0.089 (0.377)**	**-0.114 (0.160)**
**Cholesterol**	**0.159 (0.269)**	**-0.035 (0.725)**	**0.078 (0.337)**
**LDL/C**	**0.196 (0.172)**	**0.036 (0.718)**	**0.083 (0.306)**
**HDL/C**	**-0.114 (0.431)**	**-0.074 (0.460)**	**-0.082 (0.311)**
**Triglyceride**	**0.117 (0.417)**	**0.114 (0.258)**	**0.121 (0.136)**
**FAI**	**0.056 (0.701)**	**0.016 (0.872)**	**0.037 (0.649)**
**HOMA**	**0.031 (0.831)**	**0.030 (0.768)**	**0.024 (0.769)**
**QUIKI**	**0.032 (0.828)**	**0.003 (0.975)**	**0.007 (0.934)**
**G.I.R**	**0.001 (0.993)**	**0.002 (0.982)**	**-0.003 (0.976)**
**Homocysteine **	**0.045 (0.757)**	**-0.014 (0.890)**	**-0.005 (0.953)**

## Discussion

PCOS, as one of the diseases that is associated with metabolic syndrome, also may have changes in inflammation factors such as C3,CRP, interleukin-6, tumor necrosis factor-a, and lipid profiles (35, 36). We hypothesize that the PCOS state, as a low-grade chronic inflammatory state, may stimulate the immune response, increasing inflammatory factors such as CRP.

In Kaya c, Wu Y and Guzelmeric K studies were revealed that serum levels of CRP in PCOS were significantly elevated compared with age- and BMI-matched controls correlated with BMI, total cholesterol, triglyceride, low-density lipoprotein cholesterol and insulin levels and HOMA-IR . There was no correlation of CRP with parameters of PCOS such as testosterone and LH/FSH ratio (35-37).

Another study showed that in PCOS women, plasma levels of CRP were not increased when compared with age and BMI matched controls. BMI was, however, the parameter most strongly related to CRP in PCOS (38). 

The results of this study showed that the difference between the mean CRP of the two groups was not significant. Before adjusting for the age and BMI, CRP was correlated with the cholesterol, LDL and triglyceride, notably in the PCOS group. However, after the adjustment has been made for the age and BMI, the correlation was attenuated and CRP was not associated with any biochemical marker that was evaluated in this study. It seems that the biochemical changes are associated with the obesity. Thus, if the BMI is reduced, it may normalize the CRP and biochemical changes.

The impact of the lifestyle factors, in particular psychological stress, physical activity, nutrition, non smoking, normal blood pressure, metformin and the statine use has been demonstrated on CRP levels,(39-41) due to the exclusion criteria and the natural conditions of the patients in both groups. Thus, perhaps, the lack of CRP difference between the two groups could be attributed to these factors. Therefore, more detailed studies in the two groups are recommended for further investigation. It is apparent that the nutrition plays a critical role in the whole-body inflammatory response. In fact, overconsumption of highly processed foods and lack of the fruit and vegetable intake are common in North America and equivalent the increase in obesity and other inflammatory-associated diseases. Therefore lifestyle intervention is optimal for improving the body composition parameters (42, 43).

Also the results of this study showed that the obesity and metabolic alterations rather than CRP, are associated with the PCOS. The relationship between the CRP and the total homocysteine (Hcy), folate, and vitamin B12 levels is revealed as an early marker of the generalized atherosclerosis (44). Thus, it seems that to evaluate the CRP levels in the PCOS Iranian patients, a closer Look with measuring the mentioned factors is essential.
